# A DnaK(Hsp70) Chaperone System Connects Type IV Pilus Activity to Polysaccharide Secretion in Cyanobacteria

**DOI:** 10.1128/mbio.00514-22

**Published:** 2022-04-14

**Authors:** Heather J. McDonald, HoJun Kweon, Shadi Kurnfuli, Douglas D. Risser

**Affiliations:** a Department of Biology, University of the Pacific, Stockton, California, USA; Max Planck Institute for Terrestrial Microbiology

**Keywords:** cyanobacteria, DnaK(Hsp70), polysaccharide secretion, type IV pili, motility

## Abstract

Surface motility powered by type IV pili (T4P) is widespread among bacteria, including the photosynthetic cyanobacteria. This form of movement typically requires the deposition of a motility-associated polysaccharide, and several studies indicate that there is complex coregulation of T4P motor activity and polysaccharide production, although a mechanistic understanding of this coregulation is not fully defined. Here, using a combination of genetic, comparative genomic, transcriptomic, protein-protein interaction, and cytological approaches in the model filamentous cyanobacterium N. punctiforme, we provided evidence that a DnaK-type chaperone system coupled the activity of the T4P motors to the production of the motility-associated hormogonium polysaccharide (HPS). The results from these studies indicated that DnaK1 and DnaJ3 along with GrpE comprised a chaperone system that interacted specifically with active T4P motors and was required to produce HPS. Genomic conservation in cyanobacteria and the conservation of the protein-protein interaction network in the model unicellular cyanobacterium *Synechocystis* sp. strain PCC 6803 imply that this system is conserved among nearly all motile cyanobacteria and provides a mechanism to coordinate polysaccharide secretion and T4P activity in these organisms.

## INTRODUCTION

Surface motility driven by type IV pili (T4P) is ubiquitous among bacteria, including the photosynthetic cyanobacteria (1, 2). This form of movement is critical to the lifestyle of cyanobacteria, enabling dispersal, phototaxis (1, 2), the formation of complex supracellular structures (3–5), and the establishment of nitrogen-fixing symbioses with eukaryotic partners (5, 6). T4P-based motility is powered by cycles of extension and subsequent retraction of pili which adhere to the substratum or adjacent cells pulling the cell forward. Extension and retraction of the pilus are driven by the motor ATPases PilB and PilT, respectively, both of which interact with the inner membrane platform protein PilC to facilitate the addition or removal of PilA monomers from the pilus (for review see reference (7)). In cyanobacteria, two additional proteins are required for T4P extension and have been shown to interact with the base of the T4P system, Hfq and EbsA (8–10).

T4P-driven motility facilitates phototaxis in cyanobacteria, providing a mechanism for these organisms to optimize their spatial positioning in response to light (7). In many species of filamentous cyanobacteria, motility and phototaxis are only observed in specialized filaments termed hormogonia, which develop from vegetative trichomes (11). Cyanobacterial phototaxis is regulated by chemotaxis-like systems. Most of the characterized systems utilize a methyl-accepting chemotaxis protein containing one or more GAF domains, which bind bilins and are capable of directly sensing light (12). How these systems regulate the T4P motors has not been well defined. Recently, the second type of chemotaxis-like system, the Hmp system in Nostoc punctiforme, was shown to be capable of indirectly sensing light, possibly via light-driven alterations in proton motive force (9). This system regulates the association of HmpF with the T4P motors at the leading poles of cells, activating the motors and leading to directional movement. The Hmp system is the most widely conserved chemotaxis-like system among cyanobacteria (13), implying that this is a common mechanism of regulating motility in these organisms.

In several model organisms where T4P-driven motility has been investigated, the movement was shown to be dependent on the deposition of polysaccharides (14). This includes the model filamentous cyanobacterium N. punctiforme, where hormogonium polysaccharide (HPS) was shown to be required for movement (15, 16). Genes at several genetic loci have been identified as involved in the production of HPS and lectin-based analysis indicates that HPS contains fucose and possibly galactose monosaccharides. In the unicellular cyanobacterium *Synechocystis* sp. strain PCC 6803 (referred to as *Synechocystis*) polysaccharide deposition has also been posited as a requirement for T4P-driven motility, although the composition and genes responsible to produce this putative polysaccharide remain undefined (17). The production of HPS is also required for the accumulation of surface pili in N. punctiforme, implying complex coregulation of the T4P motors and HPS synthesis or secretion (18). A similar observation has been made in Myxococcus xanthus (19). Conversely, a functional T4P system was shown to be a prerequisite to produce motility-associated polysaccharides in both Myxococcus xanthus and N. punctiforme (16, 20), indicating that a complex interplay between motility-associated polysaccharides and T4P activity may be common in many bacteria.

DnaK(Hsp70) chaperone systems are found across all domains of life and are best known for their involvement in the unfolded protein response (21). In this context, DnaJ recruits unfolded proteins to DnaK, which assists in refolding the denatured protein. The activity of DnaK is dependent on cycles of ATP binding, hydrolysis, and subsequent dissociation of ADP, which are facilitated by the nucleotide exchange factor GrpE. In cyanobacteria, only a few studies have addressed the role of these chaperone systems. In the unicellular cyanobacterium *Synechocystis*, both *dnaK2* and *dnaK3* were shown to be essential and *dnaK2* was upregulated under various stress conditions (22), while in Nostoc flagelliforme *dnaK2* was shown to be involved in desiccation tolerance via enhancing PSII repair (23). However, DnaK chaperone systems have also been shown to play more specific regulatory roles in certain cellular processes. For instance, in E. coli, this system also regulates the production of curli (24). Moreover, several reports have implicated DnaK proteins in the regulation of motility in various bacteria (25–28). Here, using a combination of genetic, transcriptomic, comparative genomic, cytological, and protein-protein interaction-based approaches, we provided evidence that a chaperone system comprised of DnaK1 and DnaJ3 in N. punctiforme regulates HPS production in response to the status of T4P motor activity.

## RESULTS

### DnaK1 and DnaJ3 are cognate partners of a system required for normal hormogonium motility.

The genome of N. punctiforme encoded 5 proteins annotated as DnaK homologs, 14 DnaJ homologs, and a single GrpE homolog. Based on the nomenclature applied to putative DnaK and DnaJ proteins in the closely related cyanobacterium Nostoc flagelliform (23), we designated the N. punctiforme orthologs DnaK1-4 and DnaJ1-11 and the additional nonorthologous proteins DnaK5-6 and DnaJ12-14 (Fig. S1A). As part of an ongoing transposon mutagenic screen (29), two nonmotile isolates were identified containing a transposon insertion in *dnaK1*. The *dnaK1* gene was encoded at a locus in between *grpE* and *dnaJ11*, with all three genes in the same orientation (Fig. S1B), implying they may comprise an operon and their protein products might form a functional chaperone system. However, previously published RNAseq data (30) indicates that, while the expression of *grpE* and *dnaJ11* is static in developing hormogonia, expression of *dnaK1* is dramatically upregulated with a maximum increase in expression of ∼32-fold at 12 h post hormogonium induction (Fig. S1A and B). Enhanced expression of *dnaK1* in hormogonia appears to be most directly dependent on SigC, given that enhanced expression was reduced in both Δ*sigJ* and Δ*sigC* strains and expression of *sigC* is *sigJ*-dependent. Additionally, read coverage from total RNAseq and Cappable-seq (31) experiments indicate that, while *grpE* and *dnaJ11* were constitutively expressed in both vegetative cells and hormogonia, *dnaK1* was only expressed in hormogonia and its transcription was driven by a *sigC*-dependent promoter with a transcriptional start site −341 of the *dnaK1* start codon, which is embedded within the upstream *grpE* gene (Fig. S1B). Therefore, the expression pattern of *dnaK1* implied that it played a role specifically in hormogonia and may indicate that DnaK1 is associated with alternative DnaJ proteins in addition to, or instead of, DnaJ11.

10.1128/mbio.00514-22.1 FIG S1 Gene expression and genomic conservation of DnaK-type chaperone system components in N. punctiforme. (A) Heat maps depicting the expression of *dnaK*, *dnaJ*, and *grpE* genes in developing hormogonia of the wild-type and hormogonium-specific sigma factor mutants 0 to 18 h post hormogonium induction. Expression = log_2_(experimental strain and time point/wild-type t = 0). (B) Read map coverage from RNAseq and Cappable-Seq data for various strains and time points as indicated. (C) Heat map depicting genomic conservation of genes encoding DnaK, DnaJ, and GrpE proteins among cyanobacteria. Download FIG S1, TIF file, 1 MB. Copyright © 2022 McDonald et al. 2022 McDonald et al. https://creativecommons.org/licenses/by/4.0/ This content is distributed under the terms of the Creative Commons Attribution 4.0 International license.

To identify cognate DnaJ proteins for DnaK1, the transcriptional profiles of all annotated *dnaK* and *dnaJ* genes during hormogonium development were analyzed (Fig. S1A), and the phylogenetic co-occurrence of the N. punctiforme proteins in other cyanobacteria was investigated (Fig. S1C). Of the six *dnaK* genes, only *dnaK1* is upregulated in developing hormogonia, while the other five are either static or downregulated. Of the 14 *dnaJ* genes, 3 exhibited enhanced expression in developing hormogonia, *dnaJ3*, *dnaJ4*, and *dnaJ12*. For *dnaJ3* and *dnaJ12*, transcription was dependent on the presence of *sigJ*, while transcription of *dnaJ4* was dependent on both *sigJ* and *sigC*, indicating it is most directly regulated by *sigC*. The −10 region of the promoters for both *dnaJ3* and *dnaJ12* have also been shown to contain consensus J-Boxes (31). Based on this transcriptional data, one or more of these three DnaJ proteins may interact with DnaK1 in hormogonia.

Co-occurrence analysis of N. punctiforme DnaK and DnaJ proteins in other cyanobacteria (data derived from reference (32)) indicated that both GrpE and DnaJ11, along with DnaK2, were nearly ubiquitous among the cyanobacteria investigated, implying that these proteins comprise a system conserved in essentially all cyanobacteria (Fig. S1C). In contrast, DnaK1 showed a distinct conservation pattern with orthologs absent in the marine picocyanobacteria as well a few other phylogenetically dispersed species. Notably, the conservation pattern of DnaJ3 closely resembled that of DnaK1, while the other two DnaJ proteins upregulated in hormogonia were only present in a few other cyanobacteria (Fig. S1C). The combination of transcriptional profiles and co-occurrence for *dnaK1* and *dnaJ3* provided compelling circumstantial evidence that these proteins may be cognate partners of a chaperone system.

**FIG 1:**
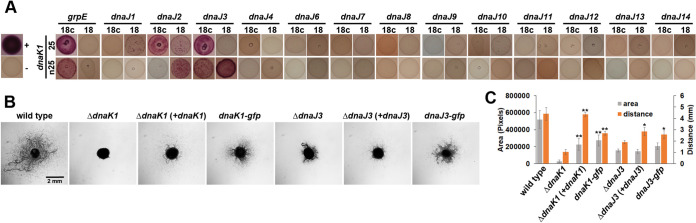
Identification of the DnaK1/J3 chaperone system. (A) BACTH analysis between DnaK1 and GrpE or DnaJs encoded in the N. punctiforme genome fused to the C (25) and N (n25) terminus of the T25 fragment or C (18c) and N (18) terminus of the T18 fragment of B. pertussis adenylate cyclase. Depicted are the results from assays on MacConkey agar. The positive-control strain (+) harbors plasmids pKT25-zip and pUT18c-zip, while the negative-control strain (−) harbors the empty vectors pKT25 and pUT18c. (B) Plate motility assays of the wild-type, deletion strains, complemented deletion strains, and strains harboring *gfp*-tagged alleles (as indicated). Images were taken at 48 h post hormogonium induction. (C) Quantification of plate motility assays depicted in (B) based on the area covered by a spreading colony and the maximal distance traveled by individual filaments. Error bars = ±1 SD. *, *P* < 0.05; **, *P* < 0.01 as determined by two-tailed Student’s *t* test between the *dnaK1-* or *dnaJ3-*deletion strains and the corresponding complemented or *gfp*-tagged allele strain, *n* = 3. All strains showed reduced motility compared to the wild-type as determined by the two-tailed Student’s *t* test, *P* < 0.05, except for maximal distance for Δ*dnaK1* (+*dnaK1*).

To provide additional experimental evidence for this hypothesis, the bacterial adenylate cyclase two-hybrid (BACTH) assay (33) was employed to probe for protein-protein interactions between DnaK1 and DnaJ proteins as well as GrpE (Fig. 1A). Of the 13 DnaJ proteins tested, a positive interaction was only detected for DnaJ2 and DnaJ3. Notably, no interaction was detected between DnaK1 and DnaJ11. We were unable to test for interaction with DnaJ5 because repeated attempts to amplify *dnaJ5* via PCR for cloning failed for unknown reasons. DnaK1 also interacted with GrpE. Collectively, the results support the hypothesis that DnaK1 and DnaJ3, along with GrpE, participate in a chaperone system active in developing hormogonium.

To confirm that *dnaK1* played a role in hormogonium development and motility and to explore a potential role for *dnaJ3* in this process, in-frame deletion strains of *dnaK1* and *dnaJ3* were created. Deletion of either *dnaK1* or *dnaJ3* drastically reduced motility in both colony spreading and time-lapse motility assays (Fig. 1B and C and Movie S1), with the reduction more pronounced in the Δ*dnaK1* strain. Notably, in time-lapse motility assays movement was observed within large aggregates but rarely for individual filaments. The mutant strains could be partially complemented by the introduction of a replicative shuttle vector containing each gene expressed from the native promoter, or by replacement of the chromosomal allele with a *gfp*-tagged variant (Fig. 1B and C). While the failure to fully complement the mutant strains could be the result of polar effects on the adjacent genes, we routinely observed that nonmotile mutants complemented in *trans* on replicative plasmids fail to restore wild-type levels of motility likely due to gene dosage effects (30). Collectively, these data implied that DnaK1 and DnaJ3 were part of a system required for normal motility in hormogonia.

10.1128/mbio.00514-22.4 MOVIE S1 Time-lapse motility assays of individual filaments from the wild-type, Δ*dnaK1,* Δ*dnaJ3* strains. Download Movie S1, AVI file, 3.6 MB. Copyright © 2022 McDonald et al. 2022 McDonald et al. https://creativecommons.org/licenses/by/4.0/ This content is distributed under the terms of the Creative Commons Attribution 4.0 International license.

### DnaK1 and DnaJ3 affect the production of hormogonium polysaccharides.

**FIG 2:**
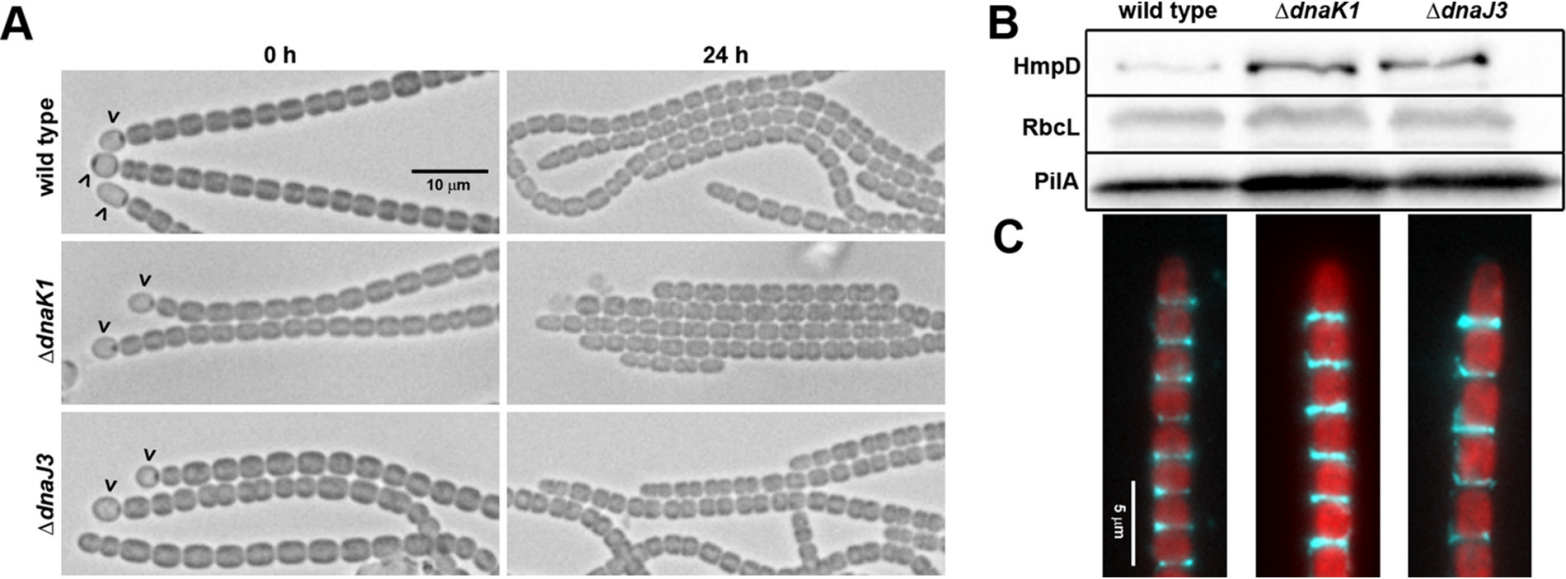
Characterization of hormogonium development in the Δ*dnaK1* and Δ*dnaJ3* strains. (A) Light micrographs of the filament morphology for the wild-type and deletion strains at 0 h and 24 h post-hormogonium induction. Carets indicate the presence of heterocysts attached to filaments. Hormogonia can be distinguished from vegetative filaments by the absence of heterocysts, smaller cell size, and the presence of tapered cells at the filament termini. (B) Immunoblot analysis of cellular HmpD, PilA, and RbcL, and (C) immunofluorescence analysis of extracellular PilA in the wild-type and deletion strains 24 h after hormogonium induction. Immunoblot analysis was performed using protein extracted from an equivalent number of cells and represents total cellular and surface-associated protein, while immunofluorescence only represents surface-associated PilA. RbcL is the large subunit of RUBISCO and serves as a protein loading control. Depicted are merged images of fluorescence micrographs acquired using a 63× lens objective from cellular autofluorescence (red) and PilA immunofluorescence (cyan).

**FIG 3:**
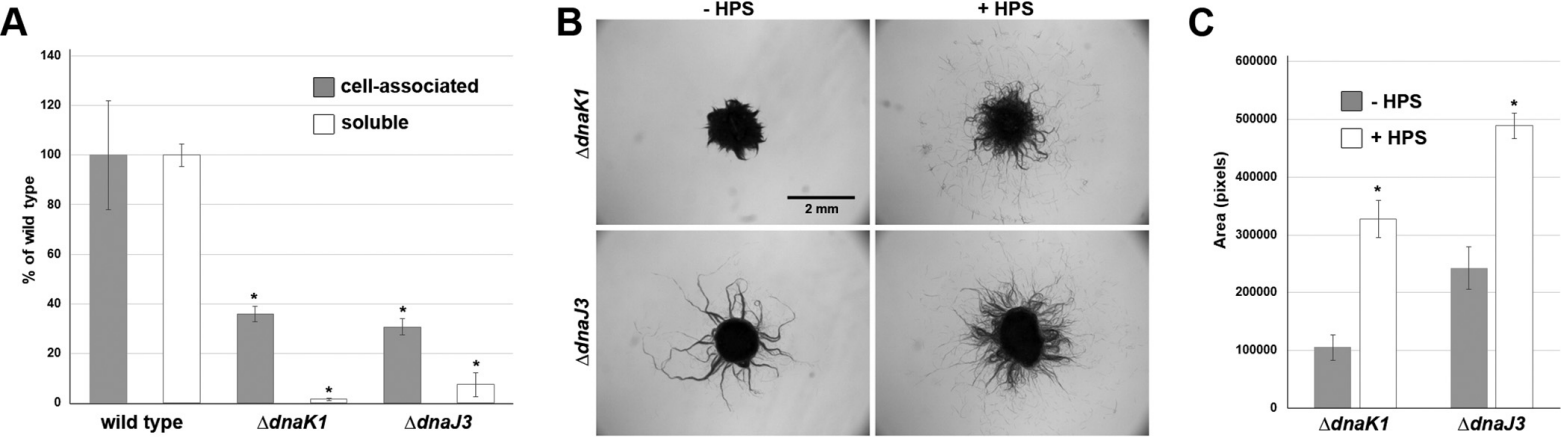
*dnaK1* and *dnaJ3* are required for the production of hormogonium polysaccharide (A) Quantification of cell-associated and soluble HPS based on fluorescent lectin staining and lectin blotting, respectively. Error bars = ±1 SD. *, *P* < 0.05 as determined by two-tailed Student’s *t* test between the wild-type and each deletion strain, *n* = 3. (B) Complementation of the *dnaK1* and *dnaJ3* deletion strains by exogenous addition of HPS. Depicted are plate motility assays of the deletion strains alone (−HPS) or supplemented with HPS from a cell-free culture medium (+HPS). Images were taken at 48 h post hormogonium induction. (C) Quantification of plate motility assays depicted in (B) based on the area covered by a spreading colony. Error bars = ±1 SD. *, *P* < 0.05 as determined by two-tailed Student’s *t* test between each deletion strain in the presence or absence of HPS, *n* = 3.

To further define the role of *dnaK1* and *dnaJ3*, several aspects of hormogonium development were investigated in the deletion strains (Fig. 2A to C). Microscopic examination of filament morphology indicated that both deletion strains produced morphologically distinct hormogonia (Fig. 2A). Immunoblot and immunofluorescence analysis of the hormogonium-specific proteins PilA and HmpD indicated slightly elevated expression of these proteins (Fig. 2B) and normal accumulation of PilA on the cell surface (Fig. 2C). However, lectin staining and lectin blotting indicated that both strains produce significantly less HPS than the wild-type strain (Fig. 3A), implying that *dnaK1* and *dnaJ3* played a critical role in regulating HPS production. Moreover, exogenous addition of HPS significantly enhanced the motility of both the Δ*dnaK1* and Δ*dnaJ3* strains (Fig. 3B and C), although not to levels seen for the wild-type strain, further supporting the hypothesis that deletion of *dnaK1* and *dnaJ3* leads to a specific defect in HPS production.

To determine if DnaK1 and DnaJ3 affected HPS production at the transcriptional level, the expression of an *hps* gene from each known *hps* locus (16) was analyzed by RT-qPCR in the Δ*dnaK1* strain (Fig. S2). All the genes tested displayed a robust increase in transcription 18 h post-hormogonium induction in both the wild-type and Δ*dnaK1* strains. Compared to the wild-type strain, only *hpsE* showed a detectable reduction in expression in the Δ*dnaK1* strain. However, this reduction was moderate, and *hpsE* expression still increased ∼16-fold in the Δ*dnaK1* strain at 18 h compared to 0 h. In contrast, expression of *hpsS* was moderately enhanced in the Δ*dnaK1* strain compared to the wild-type. Collectively, these results implied that the reduced HPS production in the Δ*dnaK1* strain was unlikely to be a result of changes in gene expression, at least for those genes currently known to be involved in HPS synthesis.

10.1128/mbio.00514-22.2 FIG S2 Expression of *hps* genes in the Δ*dnaK1* strain. The expression of various *hps* genes from known *hps* loci was determined by RT-qPCR in the wild-type and Δ*dnaK1* strains 0 and 18 h post hormogonium induction. Error bars = ±1 SD. * = *P* < 0.05 as determined by two-tailed Student’s *t* test between the wild-type and Δ*dnaK1* strain at the corresponding time point, *n* = 3. Download FIG S2, TIF file, 2 MB. Copyright © 2022 McDonald et al. 2022 McDonald et al. https://creativecommons.org/licenses/by/4.0/ This content is distributed under the terms of the Creative Commons Attribution 4.0 International license.

### DnaK1 displayed dynamic interaction with the type IV pilus motors.

**FIG 4:**
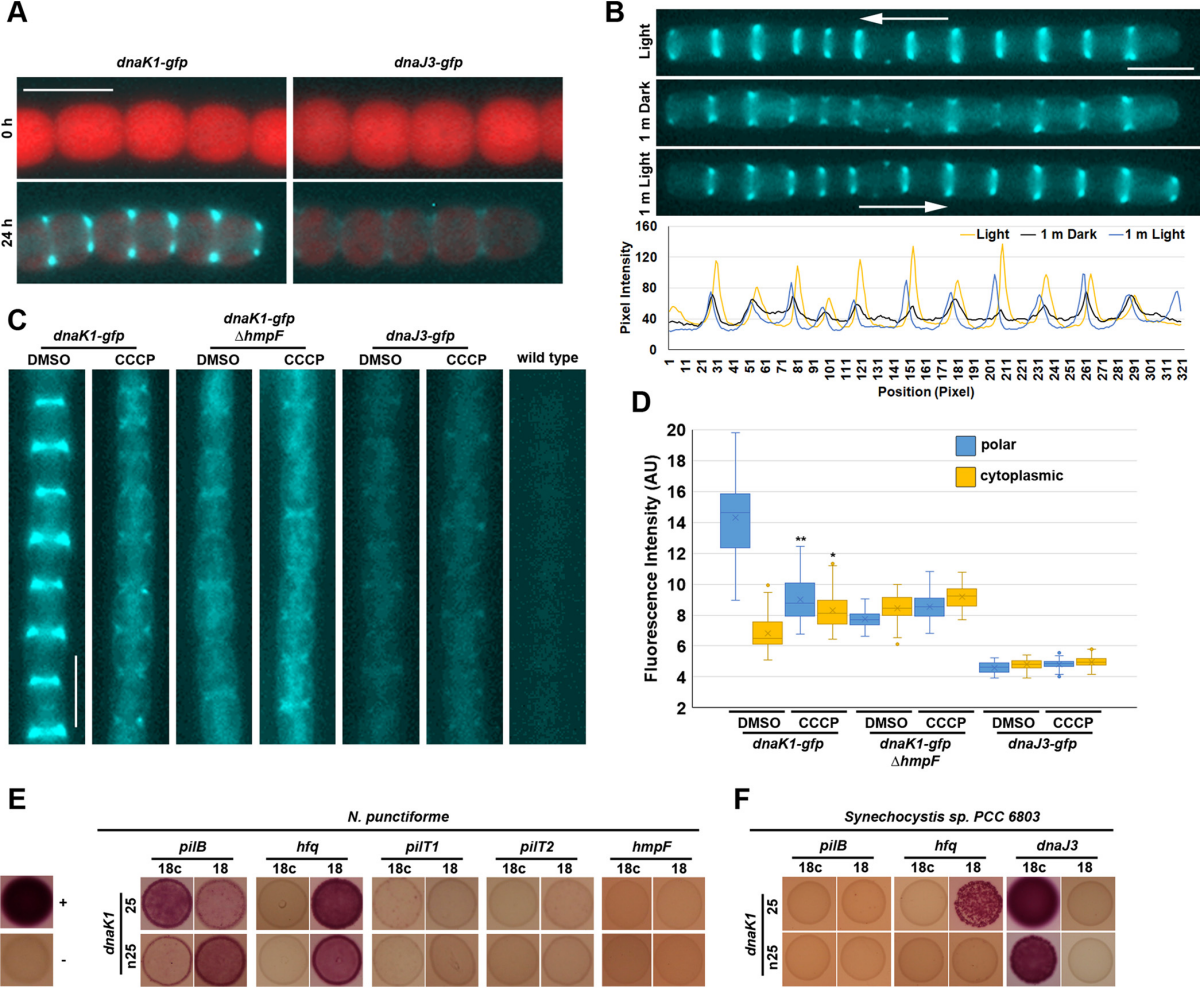
Localization of DnaK1 and DnaJ3 and interaction of DnaK1 with the T4P motor complex. (A) Fluorescence micrographs of the *dnaK1-gfp* and *dnaJ3-gfp* strains at 0 and 24 h post hormogonium induction. Depicted are merged images of fluorescence micrographs acquired using a 63× lens objective from cellular autofluorescence (red) and GFP fluorescence (cyan) from immobilized filaments. (B) Dynamic localization of DnaK1-GFP in response to light in a motile filament. Depicted are fluorescence micrographs of GFP fluorescence from a motile filament of the *dnaK1-gfp* strain preincubated in white light for 1 min (light), followed by darkness for 1 min (dark), and subsequently incubated in white light for 1 min (1 m light). Arrow indicates the direction of movement for the filament. White bar = 5 μm. The lower panel depicts the quantification of positional fluorescence intensity. Using ImageJ, a line was drawn along the length of the filament and the pixel intensity was measured at the indicated time points in the light regimen. (C) Localization of DnaK1-GFP and DnaJ3-GFP in response to treatment with CCCP or DMSO alone in a wild-type genetic background or DnaK1-GFP in a Δ*hmpF* background. Depicted are fluorescence micrographs of GFP fluorescence (cyan). White bar = 5 μm. (D) Quantification of the fraction of DnaK1-GFP or DnaJ3-GFP localized to the poles and cytoplasm. *, *P* < 0.05 as determined by two-tailed Student’s *t* test between the CCCP and DMSO treatment for each strain and position measured, *n* = 3. (E) BACTH analysis between DnaK1, and various cytoplasmic facing proteins of the T4P in N. punctiforme or (F) DnaK1 and DnaJ3 or T4P proteins from Synechocystis sp. strain PCC 6803. Proteins are fused to the C (25) and N (n25) terminus of the T25 fragment or C (18c) and N (18) terminus of the T18 fragment of B. pertussis adenylate cyclase. Depicted are the results from assays on MacConkey agar. The positive-control strain (+) harbors plasmids pKT25-zip and pUT18c-zip, while the negative-control strain (−) harbors the empty vectors pKT25 and pUT18c.

The DnaK1 ortholog in N. flagelliforme was shown to interact with the cytoplasmic membrane (23). To further explore the cellular localization of DnaK1 and DnaJ3 in N. punctiforme, the strains harboring *gfp*-tagged chromosomal alleles of *dnaK1* and *dnaJ3* were analyzed by fluorescence microscopy. For both strains, green fluorescence protein (GFP)-derived fluorescence was not detected in vegetative filaments consistent with the transcriptional data indicating these genes are transcribed specifically in hormogonia (Fig. 4A). In hormogonia, DnaK1 accumulated at the leading poles of cells in motile filaments while DnaJ3 displayed bi-polar fluorescence (Fig. 4A and B). When filaments of the *dnaK1-gfp* strain were exposed to a light regimen known to trigger dynamic localization of the T4P-associated protein HmpF, which in turn stimulates filament reversals (9), DnaK1 exhibited dynamic localization (Fig. 4B). Upon exposure to high-intensity light at 405 nm for imaging GFP followed by subsequent incubation in the dark for 1 min, a substantial fraction of the protein dissociated from the leading pole and accumulated in the cytoplasm or at the lagging cell pole such that cells displayed dimmer, bipolar fluorescence rather than bright unipolar fluorescence. When filaments were subsequently incubated in white light again for 1 min, most of the protein reaccumulated at the new leading poles of motile filaments. Immunoblot analysis with α-GFP antibodies confirmed the presence of proteins at the expected molecular weight for full-length DnaK1-GFP and DnaJ3-GFP (Fig. S3), although both strains produced lower molecular weight proteins that could have been the result of degradation of the fusion proteins. DnaK1-GFP was also much more abundant than DnaJ3-GFP, consistent with the results from fluorescence microscopy.

10.1128/mbio.00514-22.3 FIG S3 Immunoblot analysis of the wild-type, Δ*dnaK1,* and Δ*dnaJ3* strains with an α-GFP antibody. Expected molecular weight of DnaK1-GFP = ∼99 kDa, and DnaJ3-GFP = ∼63 kDa. Download FIG S3, TIF file, 1.7 MB. Copyright © 2022 McDonald et al. 2022 McDonald et al. https://creativecommons.org/licenses/by/4.0/ This content is distributed under the terms of the Creative Commons Attribution 4.0 International license.

The polar localization of HmpF was shown to be disrupted by treatment with the proton-ionophore carbonyl cyanide m-chlorophenyl hydrazine (CCCP) (9), indicating that proton motive force or some other indirect effect of CCCP treatment modulates HmpF localization. Given the similarities in the behavior of HmpF and DnaK1, the effect of CCCP treatment on DnaK1-GFP and DnaJ3-GFP was determined. Treatment with CCCP resulted in the loss of unipolar localization and a concomitant increase in cytoplasmic localization as well as low levels of bipolar accumulation for DnaK1-GFP but did not affect DnaJ3-GFP localization (Fig. 4C and D). The similarities in the localization patterns for HmpF and DnaK1 could indicate that the subcellular localization of DnaK1 was influenced by the status of the T4P motors. Alternatively, the influence of light and CCCP treatment on the behavior of DnaK1 might be due to other effects on the cell, such as depletion of ATP levels. To distinguish between these possibilities, the behavior of DnaK1-GFP was determined in a strain also harboring a deletion of *hmpF*, which is essential for activation of the T4P motors. In the *hmpF*-deletion strain, DnaK1-GFP localization was similar to that observed upon CCCP treatment in the wild-type genetic background, with high levels of cytoplasmic fluorescence and weak bipolar accumulation (Fig. 4C and D). Moreover, DnaK1-GFP no longer exhibited any changes in localization upon exposure to CCCP in the Δ*hmpF* genetic background. Both quantitation of fluorescence levels and immunoblot analysis indicated that the observed changes could not be attributed to reduced expression of the DnaK1-GFP fusion protein due to deletion of *hmpF* (Fig. 4C and D and Fig. S3). These results are consistent with a model where the status of the T4P motor influences the localization of DnaK1.

To determine whether DnaK1 interacts directly with the T4P motors, the BACTH assay was employed to test for interaction between DnaK1 and various cytoplasmic-facing T4P proteins (Fig. 4E). Interactions were detected between DnaK1 and Hfq or PilB, but not HmpF, PilT1, or PilT2. A previous study indicated that the DnaK1 ortholog in *Synechocystis* is also essential for motility (28). Therefore, the interaction between DnaK1 from *Synechocystis* and its DnaJ3, Hfq, and PilB orthologs was also tested using the BACTH assay. DnaK1 from *Synechocystis* was found to interact with both DnaJ3 and Hfq, but not PilB, indicating that this protein-protein interaction network may be at least partly conserved in cyanobacteria.

## DISCUSSION

**FIG 5:**
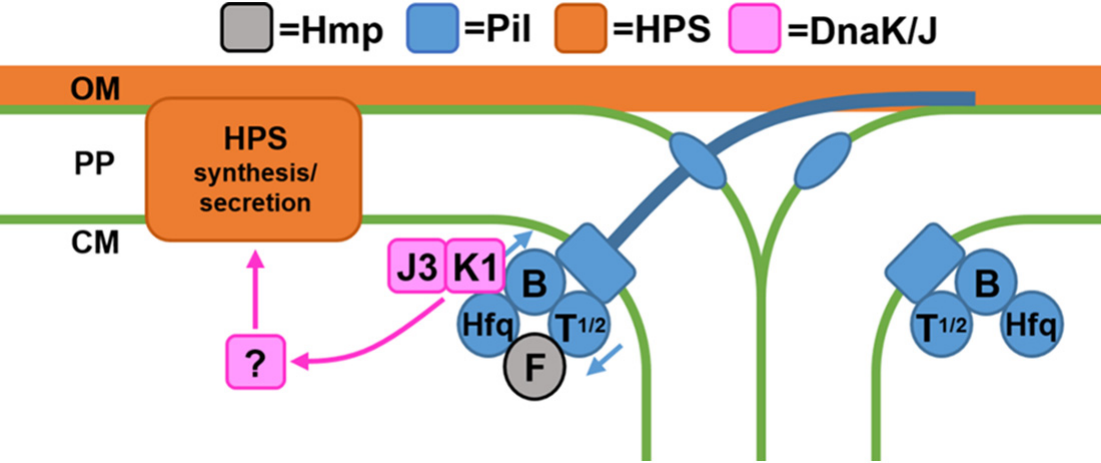
A working model of how the DnaK1/DnaJ3 chaperone system coordinates T4P activity and HPS production. Association of HmpF with the T4P motors activates cycles of pilus extension and retraction at the leading poles of cells. DnaK1 is subsequently recruited to the active motors via interaction with PilB and Hfq, where it forms a functional chaperone system influencing the folding state of an unidentified protein, which in turn influences the production of HPS. OM = outer membrane, PP = periplasm, CM = cytoplasmic membrane.

Based on the evidence provided in this study we propose a working model for how the DnaK1/J3 chaperone system influences HPS production in response to T4P activity (Fig. 5). First, HmpF associates with the T4P motors activating cycles of pilus extension and retraction. Subsequently, DnaK1 is recruited to the active motors where it forms a functional chaperone system with DnaJ3 that influences the folding state of an unidentified protein, which in turn promotes the production of HPS. Given that DnaJ2 was also found to interact with DnaK1 in the bacterial two-hybrid analysis, DnaJ2 might be involved in the system as well. Unlike *dnaK1* and *dnaJ3*, *dnaJ2* is not specifically expressed in developing hormogonia. Further experiments are needed to determine what role, if any, DnaJ2 plays in the system. In addition to the data presented in this study, several lines of evidence from previous work support this model. First, mutations that abolish T4P activity have been shown to prevent HPS secretion in N. punctiforme (18), consistent with the idea that the chaperone system is only functional when associated with active T4P motors. Second, DnaK1 was recovered in coimmunoprecipitation experiments targeting T4P proteins in both Synechococcus elongatus PCC 7942 (8) and *Synechocystis* (34). Third, a transposon-mutagenesis screen in *Synechocystis* indicated that *dnak1* is required for motility (28).

Given the wide conservation of this chaperone system in cyanobacteria and the conservation of the protein-protein interaction network in *Synechocystis*, it is likely that this system provides a ubiquitous means for cyanobacteria to couple the activity of the T4P motors to the secretion of polysaccharides. Currently, the major gap in the model is how exactly the chaperone system influences the deposition of polysaccharides. We speculate that it influences the folding state of an unknown protein which in turn regulates polysaccharide production, but further experiments are needed to identify the putative protein and lend support to this part of the model. Given the data presented here that disruption of the chaperone system does not affect transcription of known *hps* genes, we favor a posttranscriptional mechanism for the chaperone system’s influences on HPS production, although it is also possible that it influences transcription of *hps* genes that have not yet been identified. Considering that DnaK proteins have been implicated in regulating motility in several bacteria aside from cyanobacteria, such regulatory systems may be widespread outside the cyanobacterial lineage as well.

## MATERIALS AND METHODS

### Strains and culture conditions.

For a detailed description of the strains used in this study refer to Table S1. N. punctiforme ATCC 29133 and its derivatives were cultured in Allan and Arnon medium diluted 4-fold (AA/4), without supplementation of fixed nitrogen, as previously described (35), with the exception that 4 and 10 mM sucralose was added to liquid and solid medium, respectively, to inhibit hormogonium formation (36). For small scale hormogonium induction for phenotypic analysis, the equivalent of 30 μg/mL chlorophyll *a* (Chl *a*) of cell material from cultures at a Chl *a* concentration of 10 to 20 μg/mL was harvested at 2,000 × *g* for 3 min, washed two times with AA/4 and resuspended in 2 mL of fresh AA/4 without sucralose. For large-scale hormogonium induction for RT-qPCR analysis, this process was repeated but starting with the equivalent of 300 μg/mL Chl *a* of cell material and resuspension in 50 mL of fresh AA/4. For selective growth, the medium was supplemented with 50 μg/mL neomycin. Escherichia coli cultures were grown in lysogeny broth (LB) for liquid cultures or LB supplemented with 1.5% (wt/vol) agar for plates. Selective growth medium was supplemented with 50 μg/mL kanamycin, 50 μg/mL ampicillin, and 15 μg/mL chloramphenicol.

10.1128/mbio.00514-22.5 TABLE S1 Plasmids and strains used in this study. Download Table S1, DOCX file, 0.04 MB. Copyright © 2022 McDonald et al. 2022 McDonald et al. https://creativecommons.org/licenses/by/4.0/ This content is distributed under the terms of the Creative Commons Attribution 4.0 International license.

### Plasmid and strain construction.

For a detailed description of the plasmids, strains, and oligonucleotides used in this study, refer to Tables S1 and S2. All constructs were sequenced to ensure fidelity.

10.1128/mbio.00514-22.6 TABLE S2 Primers used in this study. Download Table S2, DOCX file, 0.03 MB. Copyright © 2022 McDonald et al. 2022 McDonald et al. https://creativecommons.org/licenses/by/4.0/ This content is distributed under the terms of the Creative Commons Attribution 4.0 International license.

To construct plasmids for in-frame deletion of target genes, approximately 900 bp of flanking DNA on either side of the gene and several codons at the beginning and end of each gene were amplified via overlap extension PCR (see Tables S1 and S2 for details) and cloned into pRL278 (37) as BamHI-SacI fragments using restriction sites introduced on the primers.

To construct mobilizable shuttle vectors containing *dnaK1 or dnaJ3* and its putative promoter region, the coding-region and 200 bp upstream of the TSS (31) was amplified via PCR (see Tables S1 and S2 for details) and subsequently cloned into pAM504 (38) as BamHI‐SacI fragments using restriction sites introduced on the primers.

To construct plasmid pDDR503 for replacement of the chromosomal allele of *dnaK1* with a C-terminal *gfpuv*-tagged variant, approximately 900 bp of DNA downstream of the stop codon were amplified via PCR and cloned into pSCR569 (39), as a SpeI-SacI fragment using restriction sites introduced on the primers. Approximately 900 bp of DNA upstream of the stop codon were then amplified via PCR and cloned into this plasmid as a BamHI-SmaI fragment using restriction sites introduced on the primers.

To construct plasmid pDDR506 for replacement of the chromosomal allele of *dnaJ3* with a C-terminal *gfpuv*-tagged variant, approximately 900 bp of DNA downstream of the stop codon were amplified via PCR and cloned into pSCR569 (39) as a SpeI-SacI fragment using restriction sites introduced on the primers. The coding region of *dnaJ3* and approximately 900 bp of DNA upstream of the start codon were then amplified via PCR and cloned into this plasmid as a BamHI-SmaI fragment using restriction sites introduced on the primers.

To construct plasmids encoding proteins of interest fused to either the T18 or T25 fragment of Bordetella pertussis adenylate cyclase for BACTH analysis (33, 40), the coding region of each gene was amplified via PCR and cloned into either pUT18/pUT18c or pKT25/pKNT25 using restriction sites introduced on the primers (see Table S2 for details on restriction sites used for each gene).

Generation of transposon mutants and identification of transposon insertion sites was performed as previously described (29) using plasmid pRL1063a (41). Gene deletions and allelic replacements were performed as previously described (15) with N. punctiforme cultures supplemented with 4 mM sucralose to inhibit hormogonium development and enhance conjugation efficiency (29, 36). To construct UOP176, UOP201, and UOP 202, plasmids pDDR475, pDDR501, and pDDR503 were introduced into wild-type N. punctiforme ATCC 29133, respectively. To create UOP211, plasmid pDDR424 (32) was introduced into UOP201. To construct UOP213, plasmid pDDR506 was introduced into UOP202 and maintained as a single recombinant with selection on neomycin because counterselection on 5% sucrose repeatedly failed to yield any double-recombinant colonies with the *dnaJ3-gfp* allele.

### Motility assays.

Both plate and time-lapse motility assays, including those involving exogenous complementation with HPS, were performed as previously described (18). Quantification of colony spreading by area was performed as previously described (18). To quantify the maximal distance for individual filaments, each colony was divided into equal quadrants, and the furthest distance that a filament traveled from the center of the colony was determined for each quadrant. These measurements were then averaged for each colony and repeated in triplicate.

### Immunoblot analysis.

Preparation of N. punctiforme cell material, protein extraction, and detection of PilA, RbcL, HmpD, and GFPuv by immunoblot analysis was performed as previously described (32).

### Immunofluorescence and fluorescent lectin staining.

Detection of PilA and HPS by immunofluorescence and fluorescent lectin staining was performed as previously described (32).

### Bacterial adenylate cyclase two-hybrid assays.

The bacterial adenylate cyclase two-hybrid (BACTH) assay (33, 40) was employed to probe protein-protein interaction between various proteins. BTH101 (adenylate cyclase-deficient) E. coli strains transformed with appropriate plasmids were streaked onto lysogeny broth (LB) agar plates containing 100 μg/mL ampicillin and 50 μg/mL kanamycin and incubated at 30°C for 24 h. Qualitative assays on MacConkey agar were performed as previously described (42), with several modifications as described (43).

### RT-qPCR.

RNA extraction was performed as previously described (35). 500 ng of total RNA was used to synthesize cDNA with the ProtoScript First Strand cDNA synthesis kit and random hexamer primers (New England BioLabs Inc.) following the specifications of the manufacturer, after which 1 μL of cDNA was used as a template for qPCR. Transcripts were amplified with the primer sets indicated in Table S2 using a Step One Plus real-time PCR system (Applied Biosystems) and SensiFAST SYBR No-ROX kit (Bioline) following the manufacturer’s specifications. Quantification of transcript abundance was calculated from the average of two technical replicates from each of three biological replicates using the 2^−ΔΔCT^ method (44), with expression normalized relative to *rnpB*. Primer efficiencies for each primer pair were all greater than 90%.

### Microscopy.

Light microscopy of filament morphology was performed using a Leica DME light microscope with a 40× lens objective and equipped with a Leica DFC290 digital camera controlled by micromanager imaging software (45).

Fluorescence microscopy was performed with an EVOS FL fluorescence microscope (Life Technologies) equipped with a 10× or 63× lens objective. Excitation and emission were as follows: EVOS™ light cube, GFP (AMEP4651: excitation 470 ± 22 nm, emission 525 ± 50 nm) for UEA-fluorescein labeled HPS; EVOS™ Light Cube, DAPI (AMEP4650: excitation 357 ± 44 nm, emission 447 ± 60 nm) for immunofluorescence labeled PilA; EVOS™ light cube, Nrw 405 (AMEP4857: excitation 390 ± 18 nm, emission 525 ± 50 nm) for GFPuv; and EVOS™ Light Cube, RFP (AMEP4652: excitation 531 ± 40 nm, emission 593 ± 40 nm) for cellular autofluorescence. To image immobilized filaments expressing GFP fusion proteins 5 μL of culture were placed on a dehydrated 1% agarose pad on a glass slide and overlaid with a coverslip. To image mobile filaments wet mounts were prepared using 10 μL of culture on a glass slide overlaid with a coverslip. For treatment with carbonyl cyanide m-chlorophenyl hydrazine (CCCP), 1 μL of 10 mM CCCP in DMSO, or DMSO alone was added to 1 mL of culture and incubated for 15 min. Subsequently, 5 μL of culture were placed on a hydrated 1% agarose pad containing 10 μM CCCP, or DMSO alone and overlaid with a coverslip.

Quantification of polar and cytoplasmic fluorescence derived from GFP-fusion proteins was performed using ImageJ (NIH). A line was drawn perpendicular to the long axis of the filament across the width of the cell junction or the middle of the cell for 5 contiguous cells for each of 5 filaments from 3 biological replicates and the average pixel intensity was measured for these regions.
