# Supplementary figures and images for: A DnaK(Hsp70) Chaperone System Connects Type IV Pilus Activity to Polysaccharide Secretion in Cyanobacteria
Source: mBio. 2022 Apr 14;13(3):e00514-22. doi: 10.1128/mbio.00514-22 (PMC9239167; doi:10.1128/mbio.00514-22)

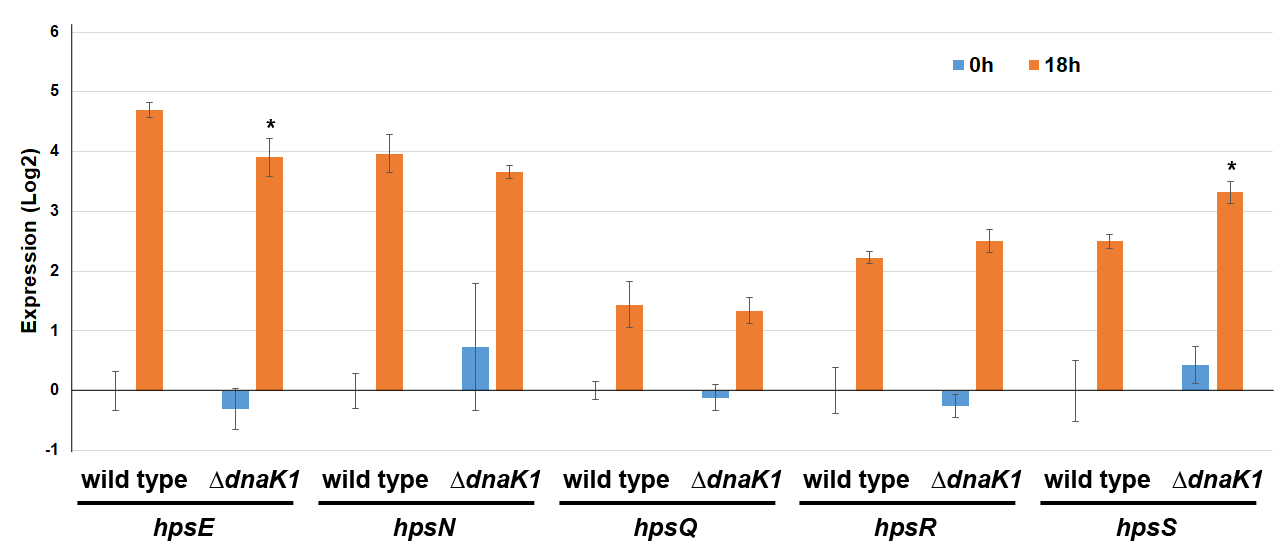

Supplement: FIG S2 [file mbio.00514-22-s0002.tif]

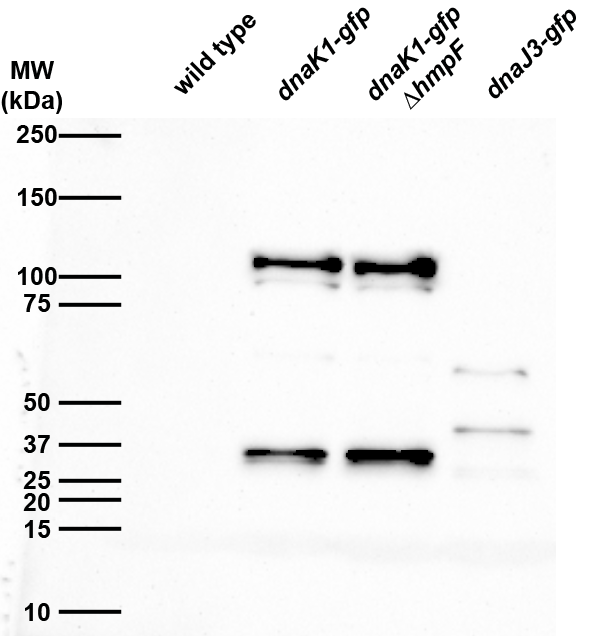

Supplement: FIG S3 [file mbio.00514-22-s0003.tif]
