# Supplementary material for: A DnaK(Hsp70) Chaperone System Connects Type IV Pilus Activity to Polysaccharide Secretion in Cyanobacteria
Source: mBio. 2022 Apr 14;13(3):e00514-22. doi: 10.1128/mbio.00514-22 (PMC9239167; doi:10.1128/mbio.00514-22)
Supplement: TABLE S1 [file mbio.00514-22-s0005.docx]

| **Table S1. Strains and plasmids used in this study** | | | |
| --- | --- | --- | --- |
| Strains | Relevant Characteristic(s) | | source |
| *Nostoc punctiforme* strains | |  |  |
| ATCC 29133 | | wild type | ATCC |
| UCD153 | | Laboratory derivative of *N. punctiforme* ATCC 29133 with reduced motility | (1) |
| UOP176 | | Δ*dnaK1* (Npun_F0122) * | This study |
| UOP202 | | Δ*dnaJ3* (Npun_F1160) | This study |
| TNM719 | | UCD153 with a Tn5-1063 insertion after nucleotide 754 of *dnaK1* | This study |
| TNM14173 | | UCD153 with a Tn5-1063 insertion after nucleotide 845 of *dnaK1* | This study |
| UOP201 | | *dnaK1-gfp* | This study |
| UOP211 | | *dnaK1-gfp,* Δ*hmpF* | This study |
| UOP213 | | *dnaJ3-gfp* single recombinant strain, neo^r^ | This study |
| Plasmids |  | |  |
| pAM504 | | Mobilizable shuttle vector | (2) |
| pRL278 | | Mobilizable suicide vector | (3) |
| pRL1063a | | Suicide vector carrying Tn5-1063, a Tn5 derivative transposon | (4) |
| pSCR569 | | Mobilizable suicide vector for C-terminal *gfpuv* translational fusions | (5) |
| pDDR475 | | Suicide vector for in-frame deletion of *dnaK1* [1-4] † | This study |
| pDDR501 | | Suicide vector for in-frame deletion of *dnaJ3* [5-8] | This study |
| pDDR424 | | Suicide vector for in-frame deletion of *hmpF* | (6) |
| pDDR503 | | Suicide vector for allelic substitution of *dnaK1* with *dnaK1-gfpuv* [4,9-11] | This study |
| pDDR506 | | Suicide vector for allelic substitution of *dnaJ3* with *dnaJ3-gfpuv* [5,8,12,13] | This study |
| pDDR507 | | Shuttle vector containing *dnaK1* and the 540 bp 5’ to the start codon [14-15] | This study |
| pDDR508 | | Shuttle vector containing *dnaJ3* and the 239 bp 5’ to the start codon [16-17] | This study |
| pEZ105 | | pUT18-*hfq* | (7) |
| pEZ106 | | pUT18c-*hfq* | (7) |
| pEZ109 | | pUT18-*pilB* | (7) |
| pEZ110 | | pUT18c-*pilB* | (7) |
| pTVH105 | | pUT18-*hmpF* | (7) |
| pTVH106 | | pUT18c-*hmpF* | (7) |
| pDDR480 | | pUT18-*hfq* (ssr3341, *Synechocystis sp.* strain PCC6803) | (7) |
| pDDR481 | | pUT18c-*hfq* (ssr3341, *Synechocystis sp.* strain PCC6803) | (7) |
| pDDR486 | | pUT18-*pilT1* | (7) |
| pDDR487 | | pUT18c-*pilT1* | (7) |
| pDDR490 | | pUT18-*pilT2* | (7) |
| pDDR491 | | pUT18c-*pilT2* | (7) |
| pHJM100 | | pKT25-*dnaK1* [18-19] | This study |
| pHJM101 | | pKNT25-*dnaK1* [18-19] | This study |
| pHJM102 | | pUT18-*grpE* (Npun_F0121) [20-21] | This study |
| pHJM103 | | pUT18c-*grpE* [20-21] | This study |
| pHJM104 | | pUT18-*dnaJ1* (Npun_R0986) [22-23] | This study |
| pHJM105 | | pUT18c-*dnaJ1* [22-23] | This study |
| pHJM106 | | pUT18-*dnaJ2* (Npun_F2810) [24-25] | This study |
| pHJM107 | | pUT18c-*dnaJ2* [24-25] | This study |
| pHJM108 | | pUT18-*dnaJ3* (Npun_F1160) [26-27] | This study |
| pHJM109 | | pUT18c-*dnaJ3* [26-27] | This study |
| pHJM110 | | pUT18-*dnaJ4* (Npun_R3872) [28-29] | This study |
| pHJM111 | | pUT18c-*dnaJ4* [28-29] | This study |
| pHJM112 | | pUT18-*dnaJ6* (Npun_R5997) [30-31] | This study |
| pHJM113 | | pUT18c-*dnaJ6* [30-31] | This study |
| pHJM114 | | pUT18-*dnaJ7* (Npun_R5579) [32-33] | This study |
| pHJM115 | | pUT18c-*dnaJ7* [32-33] | This study |
| pHJM116 | | pUT18-*dnaJ8* (Npun_R1936) [34-35] | This study |
| pHJM117 | | pUT18c-*dnaJ8* [34-35] | This study |
| pHJM118 | | pUT18-*dnaJ9* (Npun_R6085) [36-37] | This study |
| pHJM119 | | pUT18c-*dnaJ9* [36-37] | This study |
| pHJM120 | | pUT18-*dnaJ10* (Npun_F0150) [38-39] | This study |
| pHJM121 | | pUT18c-*dnaJ10* [38-39] | This study |
| pHJM122 | | pUT18-*dnaJ11* (Npun_F0123) [40-41] | This study |
| pHJM123 | | pUT18c-*dnaJ11* [40-41] | This study |
| pHJM124 | | pUT18-*dnaJ12* (Npun_F0151) [42-43] | This study |
| pHJM125 | | pUT18c-*dnaJ12* [42-43] | This study |
| pHJM126 | | pUT18-*dnaJ13* (Npun_F2991) [44-45] | This study |
| pHJM127 | | pUT18c-*dnaJ13* [44-45] | This study |
| pHJM128 | | pUT18-*dnaJ14* (Npun_F5908) [46-47] | This study |
| pHJM129 | | pUT18c-*dnaJ14* [46-47] | This study |
| pDDR509 | | pKT25-*dnaK1syn* (sll0058, *Synechocystis sp.* strain PCC6803) [48-49] | This study |
| pDDR510 | | pKNT25-*dnaK1syn* (sll0058, *Synechocystis sp.* strain PCC6803) [48-49] | This study |
| pDDR511 | | pUT18-*pilBsyn* (slr0063, *Synechocystis sp.* strain PCC6803) [50-51] | This study |
| pDDR512 | | pUT18c-*pilBsyn* (slr0063, *Synechocystis sp.* strain PCC6803) [50-51] | This study |
| pDDR513 | | pUT18-*dnaJ3syn* (sll1384, *Synechocystis sp.* strain PCC6803) [52-53] | This study |
| pDDR514 | | pUT18c-*dnaJ3syn* (sll1384, *Synechocystis sp.* strain PCC6803) [52-53] | This study |

* locus tag and genes derived from *Synechocystis* sp. strain PCC6803 rather than *N. punctiforme* denoted in parentheses

† numbers in brackets correspond to primers used to construct plasmid. Detailed information on primers can be found

in Table S2

**Supplemental References**

1. Campbell EL, Summers ML, Christman H, Martin ME, Meeks JC. 2007. Global gene expression patterns of *Nostoc punctiforme* in steady-state dinitrogen-grown heterocyst-containing cultures and at single time points during the differentiation of akinetes and hormogonia. J Bacteriol 189:5247-5256. JB.00360-07 [pii].

2. Wei TF, Ramasubramanian TS, Golden JW. 1994. *Anabaena* sp. strain PCC 7120 *ntcA* gene required for growth on nitrate and heterocyst development. J Bacteriol 176:4473-4482.

3. Cai YP, Wolk CP. 1990. Use of a conditionally lethal gene in *Anabaena* sp. strain PCC 7120 to select for double recombinants and to entrap insertion sequences. J Bacteriol 172:3138-3145.

4. Wolk CP, Cai Y, Panoff JM. 1991. Use of a transposon with luciferase as a reporter to identify environmentally responsive genes in a cyanobacterium. Proceedings of the National Academy of Sciences 88:5355-5359. 10.1073/pnas.88.12.5355.

5. Risser DD, Wong FC, Meeks JC. 2012. Biased inheritance of the protein PatN frees vegetative cells to initiate patterned heterocyst differentiation. Proc Natl Acad Sci U S A 109:15342-15347. 1207530109 [pii].

6. Cho YW, Gonzales A, Harwood TV, Huynh J, Hwang Y, Park JS, Trieu AQ, Italia P, Pallipuram VK, Risser DD. 2017. Dynamic localization of HmpF regulates type IV pilus activity and directional motility in the filamentous cyanobacterium *Nostoc punctiforme*. Mol Microbiol 106:252-265. 10.1111/mmi.13761 [doi].

7. Harwood TV, Zuniga EG, Kweon H, Risser DD. 2021. The cyanobacterial taxis protein HmpF regulates type IV pilus activity in response to light. Proc Natl Acad Sci U S A 118:10.1073/pnas.2023988118. e2023988118 [pii].
