# Supplementary material for: A DnaK(Hsp70) Chaperone System Connects Type IV Pilus Activity to Polysaccharide Secretion in Cyanobacteria
Source: mBio. 2022 Apr 14;13(3):e00514-22. doi: 10.1128/mbio.00514-22 (PMC9239167; doi:10.1128/mbio.00514-22)
Supplement: TABLE S2 [file mbio.00514-22-s0006.docx]

|  | **Table S2. Primers used in this study** |  |  |
| --- | --- | --- | --- |
| Primer Name | Sequence | Primer Number | qPCR target |
| NpF0122-5'-F | ATATAGGATCCATGAAGAGTGACTCTCCAGC | 1 |  |
| NpF0122-5'-R | cctaatctatTTTTCCCATAGTCGCCAG | 2 |  |
| NpF0122-3'-F | ctatgggaaaaATAGATTAGGGATTGGGTAATG | 3 |  |
| NpF0122-3'-R | ATATAGAGCTCAGCGCGGGTTTCTGGTTC | 4 |  |
| NpF1160-5'-F | atataggatccCAATGCCCAATAACAAATGAC | 5 |  |
| NpF1160-5'-R | cttcccGAAAGACATATAGACTTTTTGCAC | 6 |  |
| NpF1160-3'-F | gtctatatgtctttcGGGAAGAAAAAATAATGGTGTATC | 7 |  |
| NpF1160-3'-R | atatagagctcCCCCAAAAGTTGGTCATAG | 8 |  |
| NpF0122-gfp-5'-F | atataggatccCAAAGCTCCCGATCGCTC | 9 |  |
| NpF0122-gfp-5'-R | atatacccgggAATCTATCGCCTCATAATCAAC | 10 |  |
| NpF0122-gfp-3'-F | atataactagtGGATTGGGTAATGGGTAATG | 11 |  |
| NpF1160-gfp-5'-R | atatacccgggATTTTTTCTTCCCACCAAACAAAC | 12 |  |
| NpF1160-gfp-3'-F | atataactagtTGGTGTATCAGCCAGCATC | 13 |  |
| PNpF0122-BamHI-F | atataggatccTCAAACCTCAATCTGATGG | 14 |  |
| NpF0122-SacI-R | atatagagctcCTAATCTATCGCCTCATAATC | 15 |  |
| PNpF1160-BamHI-F | atataggatccGGGATAATTTTGTTGCAAGAAGC | 16 |  |
| NpF1160-SacI-R | atatagagctcTTATTTTTTCTTCCCACCAAACAAAC | 17 |  |
| NpF0122-TH-BamHI-F | atataggatcccATGGGAAAAGTTATTGGAATCG | 18 |  |
| NpF0122-TH-KpnI-R | atataggtacccgATCTATCGCCTCATAATCAAC | 19 |  |
| NpF0121-TH-BamHI-F | atataggatcccATGAAGAGTGACTCTCCAG | 20 |  |
| NpF0121-TH-KpnI-R | atataggtacccgACTGTTGGCTGGACTCGAC | 21 |  |
| Npun_R0986-TH-BamHI-F | atataggatcccATGGATCTTGGAGATTGCTAC | 22 |  |
| Npun_R0986-TH-KpnI-R | atataggtacccgAAAAATTTGTTCTAAGCGTTGG | 23 |  |
| Npun_F2810-TH-BamHI-F | atataggatcccATGGTCAATTCTAAGCACG | 24 |  |
| Npun_F2810-TH-KpnI-R | atataggtacccgTATAACAGATGCTTGTGCCTC | 25 |  |
| NpF1160-TH-BamHI-F | atataggatcccATGTCTTTCAAAATAGATCGTGG | 26 |  |
| NpF1160-TH-KpnI-R | atataggtacccgTTTTTTCTTCCCACCAAACAAAC | 27 |  |
| Npun_R3872-TH-BamHI-F | atataggatcccATGTCACAGACCTTACTACC | 28 |  |
| Npun_R3872-TH-KpnI-R | atataggtacccgACACTTGACTTGAGATCCTTTTC | 29 |  |
| Npun_R5997-TH-BamHI-F | atataggatcccATGCAAAATTTGCCGAATTTCC | 30 |  |
| Npun_R5997-TH-KpnI-R | atataggtacccgACTCAGTAAATCAGCACGG | 31 |  |
| Npun_R5579-TH-BamHI-F | atataggatcccGTGCGAATTCCGCTAGATTAC | 32 |  |
| Npun_R5579-TH-KpnI-R | atataggtacccgTCTGGTGAATTGATTTACAACAG | 33 |  |
| Npun_R1936-TH-BamHI-F | atataggatcccATGGCTCGCAAAAAATCACTTC | 34 |  |
| Npun_R1936-TH-KpnI-R | atataggtacccgTCTCATTAATTCTAACAACATCTG | 35 |  |
| Npun_R6085-TH-BamHI-F | atataggatcccATGGCTGCAACCGACTTCAAAG | 36 |  |
| Npun_R6085-TH-KpnI-R | atataggtacccgCAGCTTGACTTGCTGCAAATG | 37 |  |
| Npun_F0150-TH-BamHI-F | atataggatcccATGCTTACCGAATTTGACAC | 38 |  |
| Npun_F0150-TH-KpnI-R | atataggtacccgACTTGTTTTCGGTTTGATGTATG | 39 |  |
| NpF0123-TH-BamHI-F | atataggatcccATGGCCCGCGACTATTATG | 40 |  |
| NpF0123-TH-KpnI-R | atataggtacccgCTTAAATAAATTTCCCAAAAATCCTTC | 41 |  |
| NpF0151-TH-BamHI-F | atataggatcccATGAGCGATCGCTTTGATATAAATC | 42 |  |
| NpF0151-TH-KpnI-R | atataggtacccgGATATCGCATTGCCAAAGC | 43 |  |
| Npun_F2991-TH-BamHI-F | atataggatcccATGGTTGCAGCAACCGATTTC | 44 |  |
| Npun_F2991-TH-KpnI-R | atataggtacccgTAACCGCACCTCTTCTAAG | 45 |  |
| Npun_F5908-TH-BamHI-F | atataggatcccATGCCAACTGCAAATGATTTC | 46 |  |
| Npun_F5908-TH-KpnI-R | atataggtacccgTAACGTCACTCCTTCTAATG | 47 |  |
| sll0058-TH-BamHI-F | atataggatcccATGGGCAAAGTCATTGGCATTG | 48 |  |
| sll0058-TH-KpnI-R | atataggtacccgATCGATCGCTTCATAGTCAC | 49 |  |
| slr0063-TH-BamHI-F | atataggatcccATGACATCTTCCTCCTCTTC | 50 |  |
| slr0063-TH-KpnI-R | atataggtacccgGCTAAACCGGGAAGTCATG | 51 |  |
| sll1384-TH-BamHI-F | atataggatcccATGAGCTCGTTTCCCATCAAG | 52 |  |
| sll1384-TH-KpnI-R | atataggtacccgTTTCTTTTTGCCCCCGAATAAC | 53 |  |
| qNpun_F0070-F2 | GGTAGCCAAATTCACCCTGA | 54 | hpsE |
| qNpun_F0070-R2 | TTGCCTTGAACTCTCCCAGT | 55 | hpsE |
| qNpR638-F | AGCCCATTTGGTGCATTTAG | 56 | hpsN |
| qNpR638-R | CACTTTTATGGCAGGGTGGT | 57 | hpsN |
| qNpF1388-F | GGACACCACCAAGCGTACTT | 58 | hpsQ |
| qNpF1388-R | TGTTAGTTGGTTGCGACAGG | 59 | hpsQ |
| qNpR1506-F | TAACGGTGAGGCGGATTTAC | 60 | hpsR |
| qNpR1506-R | GCGAATTGTAATTGGGCAGT | 61 | hpsR |
| qNpR5614-F | ATGCCAGACACCCAAATCTC | 62 | hpsS |
| qNpR5614-R | TTGTGGGCCCTTTGTTCTAC | 63 | hpsS |
| qNpun_r018_F2 | CACAGAAAGATACCGCCAGA | 64 | rnpB |
| qNpun_r018_R2 | ATACTGCTGGTGCGCTCTTA | 65 | rnpB |
| Tn5-seq-F | CGATGAAGAGCAGAAGTTATC | 66 |  |
| Tn5-seq-R | GGCTCTATTCAGGATAAATC | 67 |  |
| Tn5-seq-F-nest | CGTTACCATGTTAGGAGGTC | 68 |  |
